# Supplementary material for: Activities of daily living, self-efficacy and motor skill related fitness and the interrelation in children with moderate and severe Developmental Coordination Disorder
Source: PLoS One. 2024 Apr 23;19(4):e0299646. doi: 10.1371/journal.pone.0299646 (PMC11037543; doi:10.1371/journal.pone.0299646)
Supplement: Attachment — Submitted filename: Response to Reviewers.docx [file pone.0299646.s002.docx]

| **Reviewer 1** |  |
| --- | --- |
| **Reviewer comment** | **Response from author and amendments made** |
| The aim of the study is to determine musculoskeletal fitness level, the level of activities of daily living in children (ADL) as reported by their parent and self-efficacy as reported by the children with moderate and sever DCD and compare that to typically developing children (TD). Overall, the paper reads very well, is well conducted, and includes a large group of children with DCD meeting all diagnostic criteria. That physical fitness, ADL, and self-efficacy are compromised in DCD has been reported in previous studies, although not in an African country, and not related to different levels of motor impairment. However, the relation between the three concepts has hardly been researched before. I would recommend to focus more on this relation in the introduction. At present, the introduction mainly focuses on compromised physical fitness and poor self-efficacy. Research question 2 needs more justification than presently given, as the need to study ADL is hardly mentioned in the introduction. The same holds for research question 4: why is it important to study the relationship between these concepts? And what kind of relationship do you expect based on theory/previous research? The impact of the paper would increase if those 2 research questions are more thoroughly introduced. | We thank the reviewer for her valuable and detailed comments which helped to improve the paper. New information about the relationship between these concepts has been added to the abstract, intro and discussion. So, for details please see below. |
| Title/abstract: I would suggest to include the relationship between the different concepts in the title and abstract | We have adapted the title to accommodate for this comment.  Added info to the Abstract to a max of the 300 words. Small adaptation have also been made in the introduction. |
| Introduction: See above: rationale for research questions 2 and 4 is lacking | We think the rationale for the study has been described at several points in the introduction. For instance the text reads” “However, little is known, about the children’s difficulties in specific activities of daily living (ADL) and participation in ADL [17]. Moreover, the scares evidence available comes from small samples without taking into account the severity of the motor skill impairment. Poor motor coordination will hamper many ADL activities, keeping balance while putting on pants, close shirt buttons, lace shoes or drink from a cup without spilling. “Information about the impact of the severity of motor coordination level on the mastering these skills will improve the understanding of the disorder and help to define priorities for rehabilitation.”….  “It is therefore critical to identify children who lack musculoskeletal fitness prerequisites needed for sports activities at a young age.  “”To make the picture complete, reports what parent see their children actually do during everyday life and the perceived competence for perspective of the child are needed in addition to standardized motor and fitness tests as that information will be the starting point for any intervention. “ “Although, low values for health-related fitness components, participation and perceived competency have been noted in the literature, studies having data on all these components in a large group of children with verified DCD criteria mentioned in the Diagnostic and Statistical Manual of mental disorders, fifth edition (DSM-5), over the full range of low motor scores and from a non-Western background are scarce. Little work has examined the consequences of the level of motor deficiency regarding these issues. |
| Line 92: ‘needed sports’ ‘needed for sports’ | Thanks. It has been changed on line 114 |
| **Methods:** |  |
| Line 122-123: The DCDDailyQ and CSAPPA were translated into the Arabic language. Was the backward-forward method applied? | Yes, the backward-forward method was applied to translate the DCDDaily-Q and the CSAPPA into the Arabic language. The information has been added in the Study Design or Procedure of Method section on page 8 line 150. |
| Line 126: was the CSAPPA individually administered to the children? Please explain in the text | Yes, the CSAPPA was individually administered to the children. We have added the explanation to the text in the Study Design or Procedure section on page 8 line 155-159. |
| Line 141: ‘parental questionnaire’. This questionnaire has not been mentioned before. What kind of questionnaire was used? | Thank you, we have added the parental questionnaire’ and the reference: Psychometric properties of the DCDDaily-Q: a new parental questionnaire on children's performance in activities of daily living   B. W. van der Linde, J. J. van Netten, B. E. Otten, K. Postema, R. H. Geuze and M. M. Schoemaker.   Research in developmental disabilities 2014 Vol. 35 Issue 7 Pages 1711-1719 The information has been added to the Participants section for Materials and Methods page 9 line 173. |
| Line 147: how many children were selected by teachers, and how many met the diagnostic criteria for DCD? | In total 137 children were selected by teachers, and 109 children met the diagnostic criteria for DCD. The information has been added in the Participants section Materials and Methods on page 9 line 182-185. And a flowchart was added. (new figure 1) |
| DCDDailyQ and CSAPPA: please add that only Dutch/Canadian norms were available for these instruments. | Thank you for pointing this out. The information has been now also been added in the method section on page 10, 11 line 226, 227, 234 and 237. |
| Line 198-199: “Non-parametric correlations were calculated for the TD and DCD groups, separately.” Please add between which variables correlations were calculated/ | We added the variables Line 247, 248  Non-parametric correlations were calculated for activities of daily living (DCDDaily-Q), self-efficacy CSAPPA) and musculoskeletal fitness (PERF-FIT) the TD and DCD groups, separately. Significance was set at *p*< 0.05. |
| Considering the number of post-hoc tests: was a Bonferroni correction applied? | Yes, both for the parametric and non-parametric testing. “ANCOVA with post hoc pairwise comparison with Bonferroni correction, was performed to investigate differences in musculoskeletal fitness between the 3 groups. ANCOVA (QUADE) with BMI as covariate and with correction for multiple testing”. |
| **Results:** |  |
| Line 205: “The descriptive statistics revealed that sex was equally distributed between TD and DCD groups (p=0.275)”. Did you also check whether sex was equally distributed between the two DCD groups? At first glance, it seems that far more girls than boys were in the sDCD group compared to the mDCD group. | The reviewer is right. We have almost equal number of boys and girls TD and DCD group. The stats in the paper refer to the 3 group comparison. The 2group comparison has a much higher p-value (p.0.59) We have adapted to text in the following way” The descriptive statistics revealed that sex was equally distributed between TD and the two DCD groups (p=0.275).” |
| Table 1: Height- please present two figures after the full stop. | Two decimals have been added. |
| Table 2: Please add measurement units for the PERF-FIT subtests. Please add in column 3 what the p-values represent (differences between TD and DCD groups?). I was confused by the label BMI, please explain. | Thank you for pointing out this omission. The units have been added in Table 2 and that BMI values are for the covariate.  Because of the difference between groups in BMI, BMI was entered as covariate. In this column it is shown that the impact of BMI was significant on all power and agility items and also for throwing hopping/jumping.  We have also added the classification for the BMI, which shows why using this covariate was necessary. (New Figure 2) |
| The DCDDailyQ is written in different ways throughout the text (DCD-DAILY-Q; DCDDaily-Q). In the papers by Van der Linde, the author of the questionnaire, it is called the DCDDaily-Q | The name of the questionnaire was changed to reflect the way the author wrote it. |
| Line 299: “Data also suggest that the CwDCD did not estimate their adequacy correct”. This is not what the data reveal. Children with CwDCD rated their adequacy comparable to the TD group. This does not have to mean that it is incorrect. Young children may not be able to reflect on their own performance. The same statement also occurs in the discussion. Please reflect on this | The reviewer is right we have adapted the text:” Young children may not be able to reflect on their own performance”. |
| **Discussion:** |  |
| Lines 305-317: I miss a summary of the relation between the PERF-FIT and the questionnaires, where some interesting relations were revealed for the DCD group between participation/predilection and power/agility and motor skills. | Thank you for pointing out this omission we have added text to discuss this finding,  ” The relation between self-efficacy and DCDDaily-Q was different in the two DCD groups. The highest association with motor learning as reported by the parents was found with perceived enjoyment of physical education by the child in the moderate DCD group, if it took them longer to learn motor skills, they liked PE classes less. In the severe DCD group, the highest association with the time to learn skills emerged with perceived adequacy in physical education...”  “Remarkably, although low, the association of CSAPPA Predilection and total CSAPPA with PERF-FIT subscale was higher for power and agility than for the subscale Performance including catching, hopping and balance. Moreover, for the DCDDaily-Q participation the highest correlation was also found with Power and agility.” |
| Lines 318 and following: I miss a reference to the review of Rivilis (Rivilis, I, Hay J, Cairney, J, Klentrou, P, Liu J, Faught, BE. Physical activity and fitness in children with developmental coordination disorder: a systematic review. Res Dev Disabil. 2011, May-Jun; 32(3):894-910. Doi: 10.1013/j.ridd.2011.01.017. PMID: 21310588 | Thank you, we have added the reference |
| Line 323: “Flexibility was assessed using the sit-and-reach test, which measures the flexibility of hamstrings and lower back, and not overall joint flexibility (26)”. This sentence should be added to the methods section, where information of the sit-and-reach is scarce. | Line 208-215 We gave added the info to the method section.  “The sit-and-reach test was added to assess flexibility and was assessed using a standardized wooden box. Participants are required to sit with knees straight and legs together and the soles of their feet against a box. They had to bend forward and reach as far as possible, with two hands on top of each other and hold the position for at least 3 s. The best score out of 3 trials (in cm) was further analyzed. The level of the feet is used as zero, so that if children do not reach their toes the values is negative and if the past their toes positive. The sit and reach test has the advantage of allowing for a simple estimation of flexibility of hamstrings and lower back in a short amount of time (26).” |
| Line 325: “We recommend using goniometry of the most important joints, to get valid information about the level of hyper- or hypomobility (27)”. This comment comes out of the blue, as hyper-or hypomobility is not discussed before. | We have deleted this remark and adapted the sentence. |
| Line 327: What I miss in the discussion about BMI is the fact that the BMI levels of the children in the present study are still within the normal range. The text now reads as if the children with DCD are overweight, which is not the case. |   This is an important observation for us that the reviewer interpreted this sentence differently. Because in fact the children with s-DCD are overweight based of age and gender norms. We have added this info on page 13 added a figure to show the large difference between the groups. |
| Line 342: “Our data suggest that children’s motivation to participate in leisure sports is significantly influenced by their level of skill in motor performance”. This is to bold, as no causal directions were tested. Children with DCD indicated to be less motivated to participate in leisure sports. This may be due to their lower level of motor skill. | We changed the text to : Our data suggest that children’s motivation to participate in leisure sports is significantly related to their level of skill in motor performance, which was also reported by Larsen et al. [39]. This is most likely due to their lower level of motor skill. |
| Line 345-347: “In fact, in both groups the highest correlation between predilection to physical activity and participation was seen for the fitness scale of the PERF-FIT and not for the motor skill item series”. Do you have an explanation? Maybe because predilection to physical activity is related to leisure sports, which require fitness? | We have added our interpretation to the discussion  “Remarkably, although low, the association of CSAPPA Predilection and total CSAPPA with PERF-FIT subscale was higher for Power and agility than for the subscale Performance including catching, hoping and balance items. This may be because predilection to physical activity is related to leisure sports, which require fitness. Moreover, for the DCDDaily-Q participation the highest correlation was also found with Power and agility. “…  “ This might be explained by findings in an earlier study that revealed the scores on the PERF-FIT are significantly related to performance in active play [42]. Outdoor play and sport-like games in children are characterized by short periods of intensive physical activity, interspersed with short periods of reduced or less intensive activity [43]. If children have lower levels of explosive muscle power, anaerobic capacity and agility, this will lead to restrictions in participation [44, 45].? |
| I miss a discussion about the differences between TD and DCD groups regarding ADL and CSAPPA data, and how they related to previous studies. You refer to Noordstar and Volman, but not in relation to your own findings | We have added another study that used CSAPPA and one the used DCDDaily-Q . Not many studies have used the tools, besides in the respective psychometric papers. |
| Line 396:”This study showed that components of health-related fitness are not attainable for individuals with low motor competence”. I disagree. Your study shows that children with DCD have lower fitness levels, not that health-related fitness are not attainable. I do hope that these children can improve with training. | We changed to text to according to your positive suggestion: “Children with DCD have lower fitness levels, hopefully with targeted intervention sufficient levels of health-related fitness are attainable”. |
| I miss in the discussion a remark about the different boys-girls ratios in the mDCD and sDCD groups and how this may (or not) have affected your results | See above No significant differences were found in the frequency of sex over the 3 groups. |

Percentage of children per BMI classification

|  | Normal weight | Overweight |
| --- | --- | --- |
| TD | 63.5 | 21.3 |
| m-DCD | 28.5 | 8 |
| s-DCD | 8 | 70.7 |

| **Reviewer 2** |  |
| --- | --- |
| **Reviewer comment** | **Comment from researcher and amendments made** |
| 1. “Activities of daily living, self-efficacy and musculoskeletal fitness are compromised in children with developmental coordination disorder” may be changed to “Activities of daily, living, self-efficacy and musculoskeletal comparison among the children with developmental coordination disorder” | Based on suggestion of the reviewer we have changed to title to emphasize the uniqueness of our study. Activities of daily living, self-efficacy and musculoskeletal fitness and the interrelation in children with moderate and severe Developmental Coordination Disorder |
| 2.Please refer to: “In total 214 children participated in the study: 105 TDC and 109 CwDCD”. How the sample size was determined is unclear. Sample size selection may be verified using Lwanga and Leneshow (1990). Lwanga, S.K., Lemeshow, S. and World Health Organization, 1991. Sample size determination in health studies: a practical manual. World Health Organization | Given that we were interested in medium (>50) to large effect sizes of the difference between TD and DCD and taking into account that both parametric and non-parametric testing would be needed we aimed at safe numbers to find the differences between TD and DCD. With these numbers we were also still likely to find differences in case of the analysis with 3 groups if effects were large. We have added the power calculation to the paper. |
| 3. Author may provide flow diagram research methodology steps for sample recruitment and follow up for more clarity | Thank you for this suggestion.  A flow diagram of the research methodological steps for sample recruitment has been added for more clarity as suggested by the Reviewer; Fig 1: Selection of children and tests administered, has been added to the Participants section. |
| 4. The legend shown below Table 1 may be changed, M-DCD may be changed to m-DCD, similarly S-DCD may be changed to s-DCD to make uniform throughout the manuscript | The legends were changed as suggested p.11- Table 1 |
| 5. Table 2 may be properly formatted to identify the heading of Power and Agility, Motor item series, and Flexibility | The headings were written in bold to identify them clearly p.12- Table 2 |
| 6. In Table 2 the heading F may be F-value, legend and last column entry “all sign” may be “all significant” since “all sign” has misleading meaning, similarly “TD-DCD sign” may be “TD-DCD significant”. | The table entries were changed as suggested p.12- Table 2 |
| 7. Table 3 first column suitable heading may be provided for instance. Test description. The dF2,211 may be added at the table bottom. The degree of freedom (DF) may be used throughout the manuscript. | We have added the headings and taken the Df out |
| 8. Table 4 first two columns may be suitably named as DCD types and Statistical parameters respectively | We have added info. |
| 9. Notes below the Tables may be suitably changed to small font size or maybe in Italics. All notes must be below adjoining the table. | The notes below the tables were changed to a smaller font size |
| 10. Some typos may be checked for instance “table 3” should be “Table 3”, “Table 4, gives…” should be “Table 4, gives…” and “predilection of the children (Table 5)” should be “predilection of the children (please refer to Table 5).” “by Larsen et al. (29)” should be “by Larsen et al. (29).” | The corrections were made as suggested p.15 Line 297  p.16 Line 322  p.22 Line 413 |
| 11. Table header for Table 5 may be suitably changed. The values of Spearman’s rho are shown in the last three columns. The first two columns must be suitably named. | Thank you for this suggestion it has been changed accordingly |
| 12. Table header for first two columns of Table 6 may be suitably added. | Headers have been added |
| 13. Asper Jelsma et al. (2013), “In general the motor performance and joint mobility are not related in DCD”. Hence recommending the use of goiniometry may be disjoint here.  Jelsma, L.D., Geuze, R.H., Klerks, M.H., Niemeijer, A.S. and Smits-Engelsman, B.C. 2013. The relationship between joint mobility and motor performance in children with and without the diagnosis of developmental coordination disorder, BMC pediatrics, 13, pp.1-8. | We have taken this out and adapted the sentence. Added info from the Jelsma paper.  “We found lower flexibility in the CwDCD, while Jelsma et al. [36] reported higher prevalence of hypermobility in the DCD-group. Most likely this is caused by the different outcomes used; Beighton score versus Sit and Reach. To get valid information about the level of hyper- or hypomobility it is recommended to measure range of motion of the most important joints [37]. “ |
| 14. Some recommendations put forward by Mandich et al., (2001) as “Skill acquisition through evidence-based practices” Interventions leading to functional outcomes etc. may also be discussed  Mandich, A.D., Polatajko, H.J., Macnab, J.J. and Miller, L.T., 2001. Treatment of children with developmental coordination disorder: What is the evidence? Physical & occupational therapy in pediatrics, 20(2-3), pp.51-68. | Thank you for these suggestions.  We decided that explicit discussion of different forms of intervention was outside the scope of this paper. We therefore referred to the international DCD guideline, in which more recent studies are summarized and rated for their level of evidence. |
| 15. Some similar studies related to PERF-FIT, DCD may be compared with the present studies:   - Smits-Engelsman, B., Neto, J.L.C., Draghi, T.T.G., Rohr, L.A. and Jelsma, D., 2020. Construct validity of the PERF-FIT, a test of motor-skill-related fitness for children in low resource areas. Research in developmental disabilities, 102, p.103663 - Girish S, Raja K, Kamath A. Prevalence of developmental coordination disorder among mainstream school children in India. Journal of pediatric rehabilitation medicine. 2016 Jan 1:9(2):107-16. - Van der Linde, B.W., Van Netten, J.J., Otten, B., Postema, K., Geuze, R.H., and Schoemaker, M.M., 2013. Development and psychometric properties of the DCDDaily: a new test for clinical assessment of capacity in activities of daily living in children with developmental coordination disorder. Clinical Rehabilitation, 27(9), pp.834-844. | Thank you for pointing out some interesting references. I have read the Girish S, paper with interest but couldn’t find the relation with our study, since it is a very well performed study into the prevalence of DCD, but comparison between different tools or different subgroups are not made. We had already added info from the two Van der Linde studies in the paper and have added a sentence about a comparison to the Brazilian study. “In line with the finding of Smits-Engelsman et al., in a group of Brazilian children, the present study confirmed how important it is for clinicians to follow up on skill-related fitness to address potential future adverse health effects.” |
| 16. As authors all relevant data are within the manuscript and its supporting information files. However, no data file is uploaded. Authors also need to upload DCD Daily-Questionnaire and Children’s Self-perceptions of Adequacy in and Predilection for Physical Activity Questionnaire | I don’t think we should upload questionnaires that are not ours, only the data we collected with those tools. We had permission to use the CSAPPA tool but not to publish them.  The DCDdaily_Q is available online  http://dcddaily.com/assets/manual_dcddaily-q_feb2018.pdf |
