# Supplementary material for: Activities of daily living, self-efficacy and motor skill related fitness and the interrelation in children with moderate and severe Developmental Coordination Disorder
Source: PLoS One. 2024 Apr 23;19(4):e0299646. doi: 10.1371/journal.pone.0299646 (PMC11037543; doi:10.1371/journal.pone.0299646)
Supplement: Attachment — Submitted filename: Revision Response to Reviewers 2.docx [file pone.0299646.s003.docx]

| **Reviewer 1** |  |
| --- | --- |
| **Reviewer comment** | **Response from author and amendments made** |
|  | We thank the reviewer for her valuable and detailed comments which helped to improve the paper. |
| Abstract:  Slow motor learning was associated with lower perceived enjoyment in physical education in the moderate DCD group, and with lower perceived adequacy in physical education in the severe DCD group.  Children with DCD participate and enjoy physical activity less than their peers. This combination of lower participation, lower predilection to physical activity …. | Thanks. We have changed the sentence: “Slow motor learning was associated with perceived enjoyment of physical education in the moderate DCD group, and with perceived adequacy in physical education in the severe DCD group.” By “Slow motor learning was associated with lower perceived enjoyment in physical education in the moderate DCD group, and with lower perceived adequacy in physical education in the severe DCD group.” |
| Introduction:  Line 87: “Our current study extends on Cairney’s work [15], who reported that children with DCD had lower levels of generalized than TD children. “ A word is missing after generalized. | Thanks. We have added the words missing: “self-efficacy regarding physical activity” and changed the sentence: “Our current study extends on Cairney’s work [15], who reported that children with DCD had lower levels of generalized self-efficacy regarding physical activity than TD children.” |
| **Methods:** |  |
| Line 182: “In total 137 children were selected by teachers, and 141 children met the diagnostic criteria for DCD.” Typo? 141 is more than 137. | Thanks. We have corrected the missing: “In total 167 children were selected by teachers, and 141 children met the diagnostic criteria for DCD.” |
| Line 213: “The level of the feet is used as zero, so that if children do not reach their toes the values is negative and if the past their toes positive.” Change into: the value is negative; if they reach past their toes. | Thank you, we have changed the sentence as suggested by the reviewer: “The level of the feet is used as zero, so that if children did not reach their toes the values is negative and if they reached past their toes the value is positive.” |
| Line 226: “Norms are available based a Dutch and Spanish sample.” Change into: based upon | Thank you, we have changed the sentence: “Norms are available based on a Dutch and a Spanish sample.” By “Norms are available based upon a Dutch and a Spanish sample.” |
| Line 248: “Non-parametric correlations were calculated for activities of daily living (DCDDaily-Q), self-efficacy CSAPPA) and musculoskeletal fitness (PERF-FIT) the TD and DCD groups, separately.” Change into: were calculated between | Thank you, we have changed the sentence: “Non-parametric correlations were calculated for activities of daily living (DCDDaily-Q), self-efficacy CSAPPA) and musculoskeletal fitness (PERF-FIT) the TD and DCD groups, separately.” By “Non-parametric correlations were calculated between activities of daily living (DCDDaily-Q), self-efficacy CSAPPA) and musculoskeletal fitness (PERF-FIT) for the TD and DCD groups, separately.” |
| Line 264: “Fig 2. Percentage per the BMI classification” Change into: Percentage per BMI classification. | Thanks. We have changed the sentence: “Fig 2. Percentage per the BMI classification” by “Fig 2. Percentage per BMI classification.” |
| **Discussion:** |  |
| Line 416:” In the severe DCD group, the highest association with the time to learn skills emerged with perceived adequacy in physical education” Please add: the longer it took them to learn motor skills, the lower their perceived adequacy in physical education. | Thanks. We have changed the sentence: “In the severe DCD group, the highest association with the time to learn skills emerged with perceived adequacy in physical education.” by “In the severe DCD group, the highest association with the time to learn skills emerged with perceived adequacy in physical education. the longer it took them to learn motor skills, the lower their perceived adequacy in physical education. The longer it took them to learn motor skills, the lower their perceived adequacy in physical education.” As suggested by the reviewer. |
| Line 467:” Moreover, for the DCDDaily-Q participation the highest correlation was also found with Power and agility.” Please add direction: children who participated more had better scores for power and agility? | Based on a suggestion of the reviewer we have added the sentence: “Indeed, children who participated more in physical activities had better ability to rapidly change direction.” |

| **Reviewer 2** |  |
| --- | --- |
| **Reviewer comment** | **Comment from researcher and amendments made** |
| 1) ‘Materials and Methods Study Design or Procedure’ may be changed to under section ‘Materials and Methods Study Design Procedure’ | Based on the suggestion of the reviewer we have changed “Study Design or Procedure” to “Study Design and Procedure”. Line 144. |
| 2) There are some typos may be corrected for instance:  a) components; should be components: | Thank you for this suggestion. Line 73.  We have changed “components;” to “components:” |
| b) ‘not feel adequate in physical fitness tasks, such’ should be ‘not feel adequate for physical fitness tasks such’ | Thanks. We have changed “not feel adequate in physical fitness tasks, such” to “not feel adequate for physical fitness tasks such”. Line 79. |
| c) ‘who lack musculoskeletal fitness prerequisites needed sports should be ‘who lack the musculoskeletal fitness prerequisites needed for sports’ | Thanks. We have changed the sentence: “who lack musculoskeletal fitness prerequisites needed sports” by “who lack the musculoskeletal fitness prerequisites needed for sports”. Line 115. |
| d) To make the picture complete, reports what parent see should be To make the picture complete, reports of what parents see | The corrections were made as suggested in Line 116.  We have changed: “To make the picture complete, reports what parent see” by “To make the picture complete, reports of what parents see” |
| e) ‘ life and perspective of the child’ should be ‘life and the perspective of the child’ | Thank you for this suggestion it has been changed accordingly in line 117. |
| f) ‘Diagnostic and Statistical Manual of mental disorders,’ should be ‘Diagnostic and Statistical Manual of Mental Disorders,’ | Thank you for this suggestion it has been changed accordingly in lines 122-123. |
| g) ‘Sit-and-reach test was added as measure of flexibility.’ should be ‘A sit-and-reach test was added as a measure of flexibility.’ | Thank you for these suggestions. Line 209.  We have changed “Sit-and-reach test was added as measure of flexibility.” by “A sit-and-reach test was added as a measure of flexibility.” |
