# Supplementary material for: Activities of daily living, self-efficacy and motor skill related fitness and the interrelation in children with moderate and severe Developmental Coordination Disorder
Source: PLoS One. 2024 Apr 23;19(4):e0299646. doi: 10.1371/journal.pone.0299646 (PMC11037543; doi:10.1371/journal.pone.0299646)
Supplement: Attachment — Submitted filename: Response to Reviewers 3.docx [file pone.0299646.s004.docx]

| **Reviewer 2** |  |
| --- | --- |
| **Reviewer comment** | **Response from author and amendments made** |
|  | We thank the reviewer for her valuable and detailed comments which helped to improve the paper. |
| 'To determine musculoskeletal fitness level ..' should be 'To determine musculoskeletal fitness levels ..' | Thanks. We have changed “level” By “levels” on page 2, line 22.. |
| 'Lastly, the relation physical fitness, ..' should be 'Lastly, the relation of physical fitness, ..' | Thanks. We have changed the sentence: “'Lastly, the relation physical fitness, .” By “'Lastly, the relation of physical fitness” in page 2, line 25. |
| 'Key words' should be 'Keywords' | Thanks. We have changed “Key words” By “'Keywords” |
| 'By definition, CwDCD have ..' should be 'By definition, CwDCD has ..' | Because CwDCD stance for children with DCD, this a plural. To avoid confusion, we have written it full out throughout the text. |
| 'CwDCD face evident motor difficulties ..' should be 'CwDCD faces evident motor difficulties ..' | Because CwDCD stance for children with DCD this a plural. To avoid confusion, we have written it full out throughout the text. |
| 'on the mastering these skills.. ' should be 'on mastering these skills... ' | Because CwDCD stance for children with DCD this a plural. To avoid confusion, we have written it full out throughout the text. |
| '...that CwDCD participate less in physical activities ...' should be '...that CwDCD participates less in physical activities ...' | Because CwDCD stance for children with DCD this a plural. To avoid confusion, we have written it full out throughout the text. |
| '...on their observation on the playground...' should be '...on their observations on the playground...' | Thanks. We have changed the sentence: “on their observation on the playground” By “on their observations on the playground” in page 9, line 182. |
|  |  |
| '...they did not estimate their adequacy correct. ' should be 't....hey did not estimate their adequacy correctly. | Thanks. We have changed the sentence: “...they did not estimate their adequacy correct” By “they did not estimate their adequacy correctly ” in page 19 line 388. |

| **Reviewer 3** |  |
| --- | --- |
| **Reviewer comment** | **Comment from researcher and amendments made** |
| GENERAL |  |
| The study's objective focuses on musculoskeletal fitness in DCD and its association with the level of ADL perceived by parents and self-efficacy as perceived by children. To enhance clarity and better align with the study's objective, it is recommended to consistently use the term "musculoskeletal fitness" throughout the document. This will help avoid potential ambiguity associated with broader terms like "health-related fitness" and "motor skill-related fitness." | Thank you for pointing this out. We have added the definitions of the used terms to the introduction and have consistently used those terms throughout the document as suggested by the reviewer. We choose to use definitions provided by  Corbin, C.B., Pangrazi, R.P. and Franks, B.D. (2000) Definition: Health, Fitness and Physical Activity. President’s Council on Physical Fitness and Sports Research Digest, 3, 1-8. The two types of physical fitness most often identified are health-related physical fitness and motor skill-related physical fitness.  Health-related physical fitness consists of those components of physical fitness that have a relationship with good health. The components are commonly defined as body composition, cardiovascular fitness, flexibility, muscular endurance, and strength. Motor skill-related fitness incorporates agility, balance, coordination, speed, power, and reaction time, reflecting the performance aspect of physical fitness [[11](https://www-sciencedirect-com.kuleuven.e-bronnen.be/science/article/pii/S0891422220300937#bib0050)]. Because we use the PERF-FIT as primary outcome, which was specifically developed as is a valid test to measure motor skill-related fitness fitness , we prefer to stick to that term (and not musculoskeletal fitness). Motor skill-related fitness incorporates motor potential to carry out physical activity. |
| Additionally, consider providing a clear operational definition of the term "musculoskeletal fitness" within the document. This adjustment ensures that the terminology aligns precisely with the study's focus and minimizes the risk of confusion among readers. | Thanks. As suggested by the reviewer, we have added a definition of the different fitness terms to the introduction section on page 4, line 71-79: . Both motor skill competency and fitness are critical for participation in active play [[7](https://www.ncbi.nlm.nih.gov/pmc/articles/PMC8411243/#bib35)]. Compared to typically developing (TD) children, children with DCD are particularly likely to exhibit reduced fitness [9, 10] The two types of physical fitness most often identified are health-related physical fitness and motor skill-related physical fitness [10].  Health-related physical fitness consists of those components of physical fitness that have a relationship with good health. The components are commonly defined as body composition, cardiovascular fitness, flexibility, muscular endurance, and strength. Motor skill-related fitness incorporates agility, balance, coordination, speed, power, and reaction time, reflecting the performance aspect of physical fitness (Caspersen, Powell, & Christenson, 1985 ). |
| Throughout the entire document, there is room for improvement in the use of English | The manuscript has been checked our English professor and we hope this has improved the text. |
| SPECIFIC |  |
| 1.INTRODUCTION |  |
| The introduction offers a thorough background on DCD. To enhance its effectiveness and improve reader comprehension, it is suggested to refine the introduction emphasizing on particular aspects of musculoskeletal fitness and its correlation with activities of daily living (ADL) and self-efficacy, coupled with a more explicit differentiation from prior studies | Thank you for this suggestion. We have added the following text parts:  “Children with DCD experience considerable difficulties controlling their body movements during functional motor tasks [7]. In some tests, like for anaerobic muscle endurance (e.g. the sit- or push-ups executed in 30 s the poor coordination of repetitive movements of the tasks as well as perceptions of poor physical fitness can have a negative impact on performance”. In page 5, line 88-92.  “Importantly, Ferguson et al. [12] showed that tasks that should have been learned implicitly during play and everyday activities (running up and down stairs, lifting a box, sit to stand from a chair) were consistently poor in children with DCD. Additionally, adequate muscle strength and endurance are important for performing many of these daily activities and to participate in sports without early fatigue [21]. “ In page 6, line 117-121.  Also, in the last full paragraph of the introduction we point out the uniqueness of out study  “Although, low values of motor skill-related fitness components, participation and perceived competency have been noted in the literature, studies having data on all these components in a large group of children with verified DCD criteria mentioned in the Diagnostic and Statistical Manual of Mental Disorders, fifth edition (DSM-5), over the full range of low motor scores and from a non-Western background are scarce. Little work has examined the consequences of the level of motor deficiency regarding these issues. Therefore, we included two groups of children with DCD, one with significant movement difficulties and one at risk for motor difficulties. In page 6-7, line 132-139. |
| 2. MATERIALS & METHODS |  |
| •Participants: |  |
| The statement regarding the identification of children with a motor coordination problem by parents or teachers (Criterion B) does not explicitly align with the DSM-5 criteria for the diagnosis of DCD. To strengthen this aspect, consider revising the statement to explicitly state how the motor coordination difficulties identified by parents or teachers, as per Criterion B, specifically impact activities of daily living or academic performance. This clarification will ensure a closer alignment with the diagnostic criteria for DCD. | Thank you for these suggestions. We have added a sentence to clarify Criterion B: “the DCDDaily-Q was used to further assess interference of the motor impairment with daily activities and/or academic achievement.” Page 9, line 184-185. |
| If the acerating criteria D is solely reliant on parent interviews without involvement of specialist, it is essential to acknowledge this as a limitation of the study. Additionally, discuss how this limitation might impact the overall validity. | We have added this recommendation to limitation section, page 26, line 552-556.  “ Moreover, concerning criteria D, intellectual or cognitive impairment, was evaluated by interviews with parents or teachers and not measured with an IQ-test. Neither were children seen by a neurologist to rule out other medical conditions that could explain impaired motor development. Thus, it might be possible that some children had unnoticed cognitive impairment or co-morbidities.” |
| If the global prevalence of DCD is estimated to be 5%, & the study identified 141 out of 167 with DCD, it does indeed raise questions about the potential overestimation. In light of this discrepancy, it is crucial for the study to thoroughly discuss and address the reasons behind the higher-than-expected prevalence in the sample. Consider examining the sensitivity and specificity of the diagnostic tools used, potential biases in the identification process, or any other factors that might have contributed to the observed prevalence. Additionally, discuss how this overestimation might impact the generalizability of the study's findings and the validity of the prevalence rates reported | Thank you for pointing this out because it gave the wrong impression. We have rewritten the selection of participants for DCD and TD children to eliminate any confusion (page 9, lines 199-203.). The prevalence of DCD estimated in the current study was 6.5 %. We have changed the sentence: “In total 167 children were selected by teachers, and 141 children met the diagnostic criteria for DCD. Finely, 214 children performed all the tests, and participated in the study: 105 TD and 109 CwDCD” into “In total 141 children were identified who met the diagnostic criteria for DCD out of 2170 children from the 6 elementary schools. TD children were matched according to age and sex to the children with DCD. Finely, 214 children performed all the tests, and participated in the study: 109 children with DCD and 105 typically developing matched controls. |
| Statistical analysis: While all the tests were deemed suitable, there is a need for clarification regarding the particular non-parametric correlation method applied in the study concerning DCDDaily-Q, CSAPPA, and PERF-FIT. | We have added that we used Spearman correlations between DCDDaily-Q, CSAPPA, and PERF-FIT. Page 12, line 270. |
| Discussion: The document features a comprehensive discussion. However, potential confusion among readers may arise due to the use of terms like "health-related" and "musculoskeletal" fitness. Therefore, it is recommended to revise the wording for clarity, and addressing the limitation of the study is advised | We have revised the wording for clarity and addressed the limitation of the study as suggested by the reviewer.  We discussed parent interviews without the involvement of a school psychologist and that a neurologic examination to rule out other medical conditions was not performed (criteria D). We also added the cultural sensitivity of MABC-2 items in the limitations section (page 26, line 547-551). |
